# *Toxoplasma gondii* modulates the host cell cycle, chromosome segregation, and cytokinesis irrespective of cell type or species origin

**DOI:** 10.1186/s13071-024-06244-2

**Published:** 2024-04-05

**Authors:** Lisbeth Rojas-Baron, Kira Senk, Carlos Hermosilla, Anja Taubert, Zahady D. Velásquez

**Affiliations:** Institute of Parasitology, Biomedical Research Center Seltersberg, Justus Liebig University of Giessen, Giessen, Germany

**Keywords:** Cell cycle, *Toxoplasma gondii*, Binucleated cells, Human and bovine cells

## Abstract

**Background:**

*Toxoplasma gondii* is an apicomplexan intracellular obligate parasite and the etiological agent of toxoplasmosis in humans, domestic animals and wildlife, causing miscarriages and negatively impacting offspring. During its intracellular development, it relies on nutrients from the host cell, controlling several pathways and the cytoskeleton. *T. gondii* has been proven to control the host cell cycle, mitosis and cytokinesis, depending on the time of infection and the origin of the host cell. However, no data from parallel infection studies have been collected. Given that *T. gondii* can infect virtually any nucleated cell, including those of humans and animals, understanding the mechanism by which it infects or develops inside the host cell is essential for disease prevention. Therefore, we aimed here to reveal whether this modulation is dependent on a specific cell type or host cell species.

**Methods:**

We used only primary cells from humans and bovines at a maximum of four passages to ensure that all cells were counted with appropriate cell cycle checkpoint control. The cell cycle progression was analysed using fluorescence-activated cell sorting (FACS)-based DNA quantification, and its regulation was followed by the quantification of cyclin B1 (mitosis checkpoint protein). The results demonstrated that all studied host cells except bovine colonic epithelial cells (BCEC) were arrested in the S-phase, and none of them were affected in cyclin B1 expression. Additionally, we used an immunofluorescence assay to track mitosis and cytokinesis in uninfected and *T. gondii*-infected cells.

**Results:**

The results demonstrated that all studied host cell except bovine colonic epithelial cells (BCEC) were arrested in the S-phase, and none of them were affected in cyclin B1 expression. Our findings showed that the analysed cells developed chromosome segregation problems and failed to complete cytokinesis. Also, the number of centrosomes per mitotic pole was increased after infection in all cell types. Therefore, our data suggest that *T. gondii* modulates the host cell cycle, chromosome segregation and cytokinesis during infection or development regardless of the host cell origin or type.

**Graphical Abstract:**

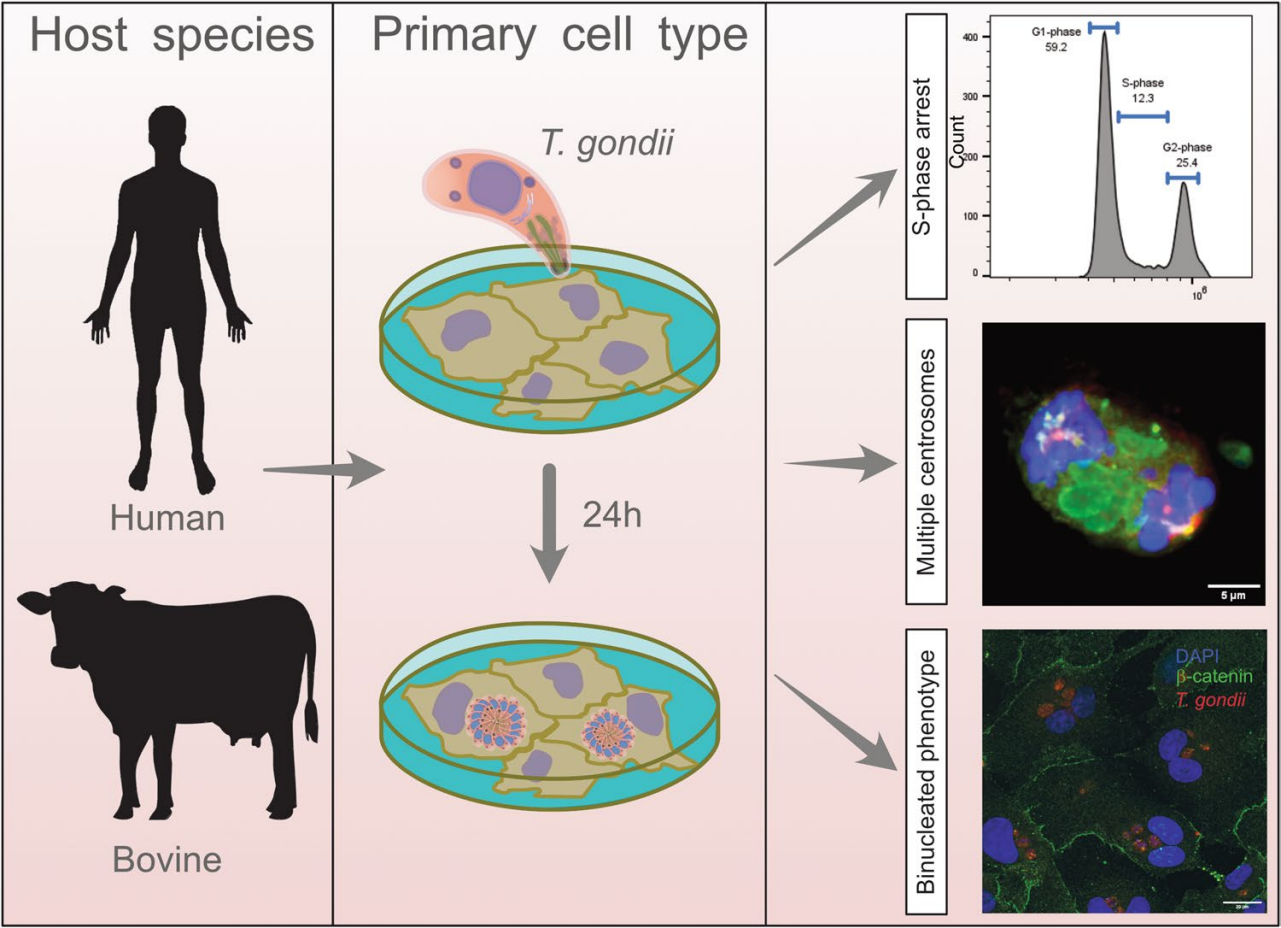

## Background

The obligate intracellular parasite *Toxoplasma gondii* is a globally spread zoonotic protozoan that causes severe health problems in both humans and animals. Prenatal infections can cause abortion or harm the progeny's welfare. Acute infections can be life-threatening in immunocompromised individuals, and several studies have postulated a correlation between latent *T. gondii* infections and neurological/psychiatric disorders in humans [1, 2].

Intracellular protozoa are well known for modulating their host cells to ensure efficient intracellular development and proliferation. As such, they have been shown to modulate a variety of host cellular functional categories, such as apoptosis, autophagy, cytoskeleton, metabolism, immunological responses and cell cycle [3–8]. Some parasite-triggered cell cycle disruption is both parasite species- and host cell type-specific. *Toxoplasma gondii*, *Leishmania *spp., *Trypanosoma cruzi* and *Encephalitozoon* spp. induce cell cycle arrest and dampen host cell proliferation, whilst *Theileria* stimulates host cell division and proliferation [9–14]. *Leishmania amazonensis* interferes early in the cell cycle with the G0/G1 phase, and *T. cruzi* triggers host cell progression to the S-phase, besides blocking host cell mitosis and impairing cytokinesis after nuclear replication [10, 12]. The host cell type seems to be important in *Plasmodium* species; for example, *P. falciparum* infections in HepG2 cells were found to affect mitosis and lead to binucleated phenotype formation without cell division [15]. However, cell cycle-dependent reactions played no role in *P. berghei*- or *P. yoelii*-infected mouse models either in vivo or in vitro. *Plasmodium falciparum*-infected primary hepatocytes provide evidence that primary cell types differ considerably from permanent cell lines in their reactions. To date, there is no information whether the control that *T. gondii* exerts on the host cell cycle is restricted to specific cell types or to only some host cell species. However, it has been shown that *T. gondii* infections shift G0/G1 cells through the S-phase in human foreskin fibroblasts (HFF) [4], but arrest cells in the G2-phase in a human trophoblast cell line and human dermal fibroblasts [3], or even both in the L6 rat myoblast cell line [16]. The G2-phase arrest in human dermal fibroblasts or a human trophoblast cell line was linked to decreased cyclin B1 abundance without alterations in other G2/M checkpoint-related proteins [3]. *T. gondii* arrest of HFF during the S-phase or G2/M was accompanied by a delayed or absent increase in cyclin A and cyclin B1, thereby indicating a missing exit from the S-phase and failure to progress [4]. *T. gondii* infections increased the proportion of polyploid cells (8n) in the murine RAW264.7 cell line, most likely reflecting DNA replication without subsequent cytokinesis [17]. All of the above suggests that *T. gondii* altered the host cell cycle based on the origin of the host or the cell type infected. However, no experiment has been carried out in parallel using different cell types and species in order to establish comparative analyses. Therefore, the current study sought to determine whether *T. gondii* regulates the host cell cycle progression based on the host cell species or type. We used commercially available primary human cells and two primary bovine cell lines in-house-isolated, controlling the passage number to maintain the original phenotype. Our findings showed that cell cycle arrest occurred in almost all primary cells excluding bovine colonic epithelial cells (BCEC). Nonetheless, *T. gondii-*induced chromosome mis-segregation and cytokinesis failure were detected in all cell types studied, suggesting that *T. gondii* modulates these two mechanisms independently of host cell origin.

## Methods

### Primary human and bovine host cells and parasite maintenance

Primary human umbilical vein endothelial cells (HUVEC, six donors in total, PromoCell), HFF (*n* = 3, PromoCell) and human small intestine epithelial cells (FHs74, American Type Culture Collection [ATCC]) were cultured at 37 °C and 5% CO_2_ following the supplier’s protocols [(media: HUVEC: Endothelial Cell Growth Medium, PromoCell; HFF: Dulbecco's modified Eagle medium [DMEM]-GlutaMAX, Gibco; FHs74: HybriCare, ATCC; BCEC: RPMI1640, Sigma; bovine small intestinal epithelial cells [BSIEC]: DMSM/F12, Gibco); see primary cell information in Table 1]. Each experiment was performed at a maximum of four passages after isolation to enable the best comparison. All cell lines were seeded at the same time and infected with the same batch of tachyzoites. *T. gondii* RH tachyzoites were maintained by serial passages in primary HFF cells (maximum passage: 10). Therefore, free-released *T. gondii* tachyzoites were harvested from HFF supernatants, pelleted (400×*g*, 12 min), counted and suspended in the corresponding medium for each host cell type. Infections were performed in sub-confluent cell layers. All experiments were performed at a multiplicity of infection (MOI) of 1:1 (cells: parasites).

**Table 1:** Primary cells used in the current study

Name of primary cell	Company	Cat. number	Lot number (donor)
HUVEC-p single donor	PromoCell	C-12250	449Z018.1
HUVEC-p single donor	PromoCell	C-12250	466Z026
HUVEC-p single donor	PromoCell	C-12250	467Z015
HUVEC-p single donor	PromoCell	C-12250	478Z023
HUVEC-p single donor	PromoCell	C-12250	486Z004
HUVEC-p single donor	PromoCell	C-12200	469Z003.1
HUVEC-p single donor	PromoCell	C-12200	473Z022.1
HFF-1	ATCC	SCRC-1041	
FHs74	ATCC	CCL-241	

### Flow cytometry-based analysis of cell cycle phases

Cellular DNA content was measured using FxCycle PI (propidium iodide)/RNAse staining solution (Invitrogen, F10797) according to the manufacturer’s instructions. Cyclin B1 quantification was performed in cells fixed with BD fixation/permeabilization solution (BD, 554714, Becton, Dickinson and Company, Heidelberg, Germany) and stained with cyclin B1-AF647 (Cell Signaling Technology, 4118). The samples were analysed using a BD Accuri C6 Plus flow cytometer (Becton–Dickinson) applying 535/5 nm excitation and emission collected in a 617/20 band-pass. Cells were gated according to their size and granularity; only morphologically intact host cells were included in the analysis. All analyses were performed in FlowJo v.10 software.

### Immunofluorescence assays

The method was performed as described by Velásquez et al. [18]. Briefly, uninfected and infected cells were fixed with paraformaldehyde (PFA, 4%, 15 min, room temperature [RT]), washed with phosphate-buffered saline (PBS) and incubated in blocking/permeabilization solution (PBS with 3% bovine serum albumin [BSA], 0.1% Triton X-100; 1 h, RT). Thereafter, samples were incubated in primary antibodies (Table 2) and diluted in blocking/permeabilization solution (overnight at 4 °C in a humidified chamber). After three washes in PBS, the samples were incubated in secondary antibody solutions (Table 2; 30 min at RT and complete darkness). Cell nuclei were labelled using a DAPI-supplemented mounting medium (Fluoromount-G, Thermo Fisher).

**Table 2:** Primary and secondary antibodies used in the western blot (WB) and immunofluorescence assays

Antigen	Company	Cat. number	Origin/reactivity	Dilution
Primary antibodies immunofluorescence assay				
Β-catenin	Abcam	Ab32572	Rabbit	1:200
*Toxoplasma gondii*	Thermo Fisher	PA1-7256	Goat	1:100
Primary antibodies FACS				
Cyclin B1-Alexa Fluor 647	Cell Signaling Technology	ab32053	Rabbit	1:50

Secondary antibodies				
Antigen/conjugate	Company	Cat. number	Host/target	Dilution
Goat anti-mouse IgG peroxidase conjugated				
Alexa Fluor 488	Thermo Fisher	A11001	Goat/mouse	1:500
Alexa Fluor 594	Thermo Fisher	A21468	Chicken/rabbit	1:500
Alexa Fluor 647	Thermo Fisher	A21235	Goat/mouse	1:500

### Image acquisition and reconstruction

Image acquisition and reconstruction were carried out according to the description published by Velásquez et al. [18]. A ReScan confocal microscope (RCM 1.1 Visible, Confocal.nl) equipped with a fixed 50 µm pinhole size and combined with a Nikon Ti2-A inverted microscope was used to acquire fluorescence and confocal images. The RCM unit was connected to a Toptica CLE laser with the following excitations: 405/488/561/640 nm. Images were acquired using a scientific CMOS [complementary metal–oxide–semiconductor] (sCMOS) camera (pco.edge, PCO) with a CFI Plan Apochromat 60× lambda-immersion oil objective (NA 1.4/0.13; Nikon). The system was operated using NIS-Elements software (version 5.11). Images were acquired via z-stack optical series with a step size of 0.1 micron depth to cover all structures of interest within the analysed host cells. Z-series were displayed as maximum z-projections. Identical brightness and contrast conditions were applied for each data set within one experiment using Fiji software [19].

### Statistical analysis

All data are expressed as mean ± standard deviation (SD) from three independent experiments. When two groups were compared, a Mann–Whitney test was performed. When three or more experimental groups were compared, a Kruskal–Wallis one-way analysis of variance was applied. Significance was defined as *P* ≤ 0.05. All graphs and statistical analyses were performed using GraphPad Prism 9 software.

## Results

**Fig. 1:**
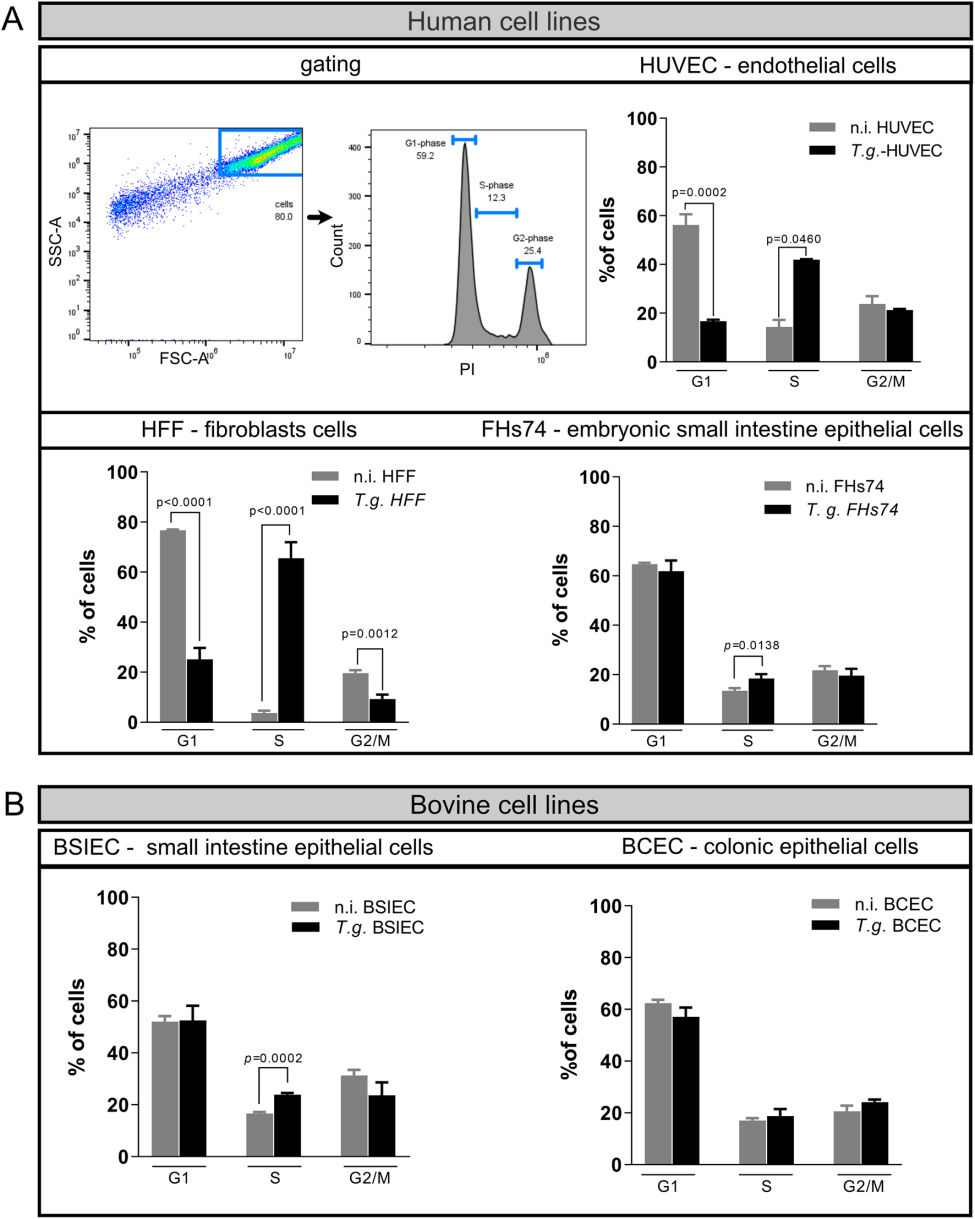
*Toxoplasma gondii* arrests primary human and bovine cells in the S-phase 24 h p.i. Three isolates of HUVEC, HFF, FHs74, BSIEC and BCEC were infected with *T. gondii* tachyzoites, and the samples were collected 24 h p.i. Fixed samples were stained with propidium iodide (PI) and analysed using FACS. Gating was performed by first selecting the cell population according to the shape and granularity. Then, the cell cycle phases were analysed using a histogram of the number of cells versus the PI signal. Cells in the first peak correspond to those in the G1-phase, the second peak to cells in the G2/M-phase, and cells in between the two peaks were cells in the S-phase. The results showed that all human cells were arrested in the S-phase (**A**), whilst only one bovine cell showed no effect on cell cycle progression before *T. gondii* infection (**B**). Graph bars represent the median ± SD of three biological replicates

*Toxoplasma gondii* has been described to modulate the host cell cycle between the G1 and G2/M phase depending on the experimental model used [3, 4, 14, 20]. Given that these experiments were performed under different experimental conditions and cell types, we wanted to know whether *T. gondii* uses this mechanism as an infection strategy or whether it is dependent on the host type or species. As host cells, we tested three human primary cells, HUVEC, FHs74 and HFF, and two primary bovine cells, BSIEC and BCEC. To avoid artefacts due to the immortalization of the cell or tumour phenotype, we worked only with primary cells, at a limit of four passages after isolation. The host cells were infected at sub-confluence, and at 24 h post-infection (p.i.) they were analysed for DNA content using fluorescence-activated cell sorting (FACS) flow cytometry. The gating process started by selecting the cell population according to their shape and granularity (Fig. 1). Cells were then plotted as the number of cells versus PI signal. The first peak represents the cells in the G1-phase, the second peak represents those cells in the G2/M-phase, and the S-phase corresponds to the cells located between the two peaks. Cells in each cell cycle phase are shown as a proportion of the total number of cells counted. DNA quantification results showed that HUVEC, HFF and FHs74 from humans and BSIEC were arrested in the S-phase after infection (Fig. 1), whereas *T. gondii* infection did not affect cell cycle progression in BCEC (Fig. 1). It should be noted that HUVEC and HFF cells showed a reduction in the number of cells in the G1-phase, and HFF also showed a reduced percentage of cells in the G2-phase (Fig. 1).

**Fig. 2:**
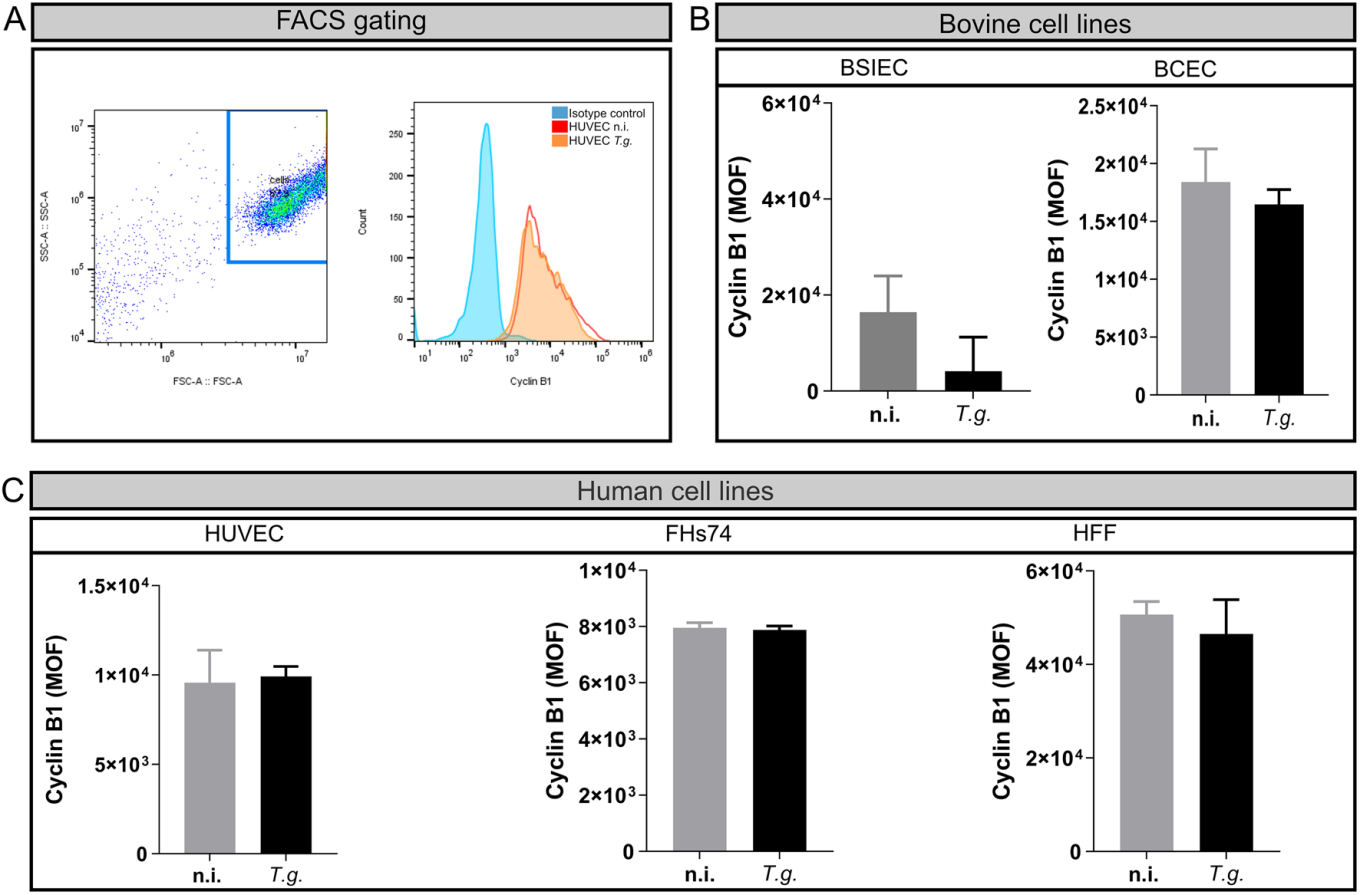
Cell cycle arrest in *T. gondii*-infected cells is independent of the mitosis checkpoint protein control cyclin B1. Three donors of HUVEC, HFF, FHs74, BSIEC and BCEC were infected with *T. gondii* tachyzoites and fixed 24 h p.i. Cells were stained against cyclin B1 and an isotype control and analysed using FACS. Gating was done first by choosing the cell population and then selecting the same number of cells in each sample (**A**). The cyclin B1 signal was analysed as the mean of fluorescence (MOF) and plotted for non-infected and *T. gondii-*infected cells. The results showed that cyclin B1 was not affected after infection in any of the cell types studied (**B**, **C**). Graph bars represent the median ± SD of three biological replicates

Cell cycle arrest in the S-phase could be explained by a blockage entry into the G2/M-phase. Therefore, we analysed the expression of one of the main regulators of the G2-to-M-phase transition, cyclin B1. Cyclin B1 accumulates throughout the S-phase, with a peak at the onset of the M-phase [21]. After that, cyclin B1 needs to be degraded to allow cells to enter mitosis. Thus, we quantified the cyclin B1 protein 24 h p.i., using FACS-based quantification. The cells were gated according to their shape and granularity and then plotted as the total number of counted cells and the cyclin B1 intensity (Fig. 2). The results showed that none of the cells modulated cyclin B1 expression after *T. gondii* infection (Fig. 2), suggesting that *T. gondii* does not arrest the host cell cycle in the S-phase due to modulation of the mitosis checkpoint protein cyclin B1.

**Fig. 3:**
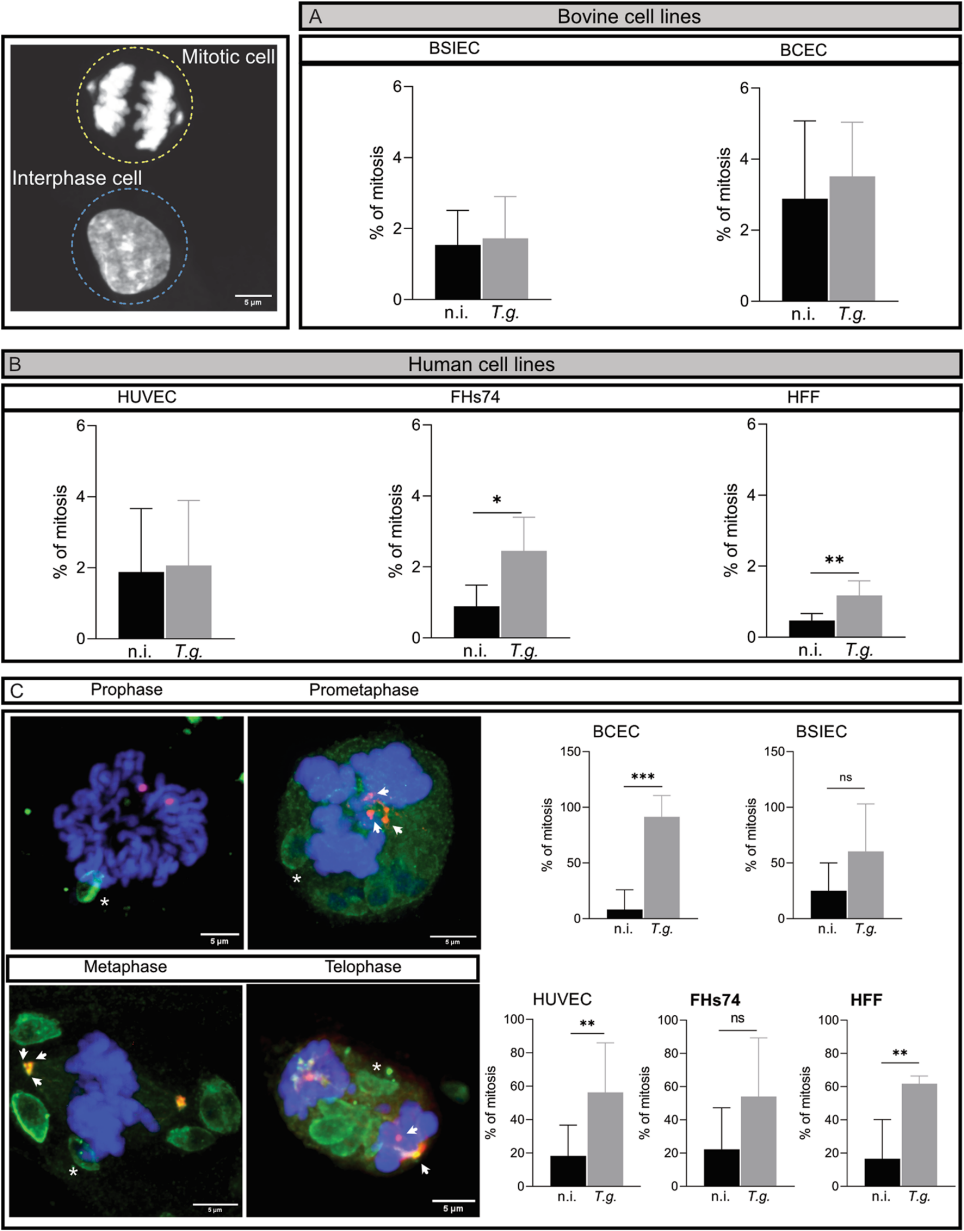
*Toxoplasma gondii* infection affects the mitosis rate and the chromosome segregation at the mitosis phase. HUVEC, HFF, FHs74, BSIEC and BCEC were infected with *T. gondii* tachyzoites, and were fixed 24 h p.i. with PFA and stained against DAPI (chromosome marker, blue), γ-tubulin (centrosome marker, magenta) and *T. gondii* (green, asterisks). The mitosis index was counted as the total number of cells facing mitosis (see scheme) related to the total number of cells in the field of view. This percentage was calculated for the bovine (**A**) and human cell lines (**B**). The results showed that no bovine cell line studied here modified its mitosis rate after infection with *T. gondii.* However, FHs74 and HFF showed an increased proportion of mitotic cells*.* **C** The mitosis phases were followed by chromosome segregation, and the results showed that all cell lines developed chromosome segregation errors mainly due to an abnormal number of centrosomes (white arrows). Thereafter, the proportion of aberrant mitosis was determined in both bovine and human cells by counting the total number of aberrant mitoses relative to the total mitotic cells (normal and aberrant). The scale bar represents 5 µm

It has been previously demonstrated that *T. gondii* infection affects mitosis progression itself, and thus we performed an immunofluorescence assay in cells infected for 24 h with *T. gondii* tachyzoites. Firstly, we quantified the percentages of cells in mitosis in the non-infected and infected monolayers (total number of cells: FHs74:1169; BSIEC:1248; BCEC:1531; HFF:6280; HUVEC:4287), with no significant differences in the mitotic index between the bovine cell lines BCEC and BSIEC and the human cell line HUVEC (Fig. 3A, B). Mitosis was evaluated from prophase (chromosome condensation) to telophase (chromosomes sets were pulled to opposite poles of the cell). On the contrary, human cell lines FHs74 and HFF increased the percentage of mitotic cells in the *T. gondii*-infected monolayer (Fig. 3B). Secondly, we quantified the aberrant mitotic cells in all human and bovine cell lines. Fixed cells were stained for chromosomes (DAPI, blue), centrosome (γ-tubulin, red) and *T. gondii* tachyzoites (green; Fig. 3C). Aberrant mitoses were detected in all phases of mitosis, as shown in Fig. 3C. We observed prometaphases or metaphases with more than two centrosome poles (Fig. 3C**,** white arrows). Similar results were observed in telophase, suggesting that *T. gondii* affects the centrosome number and therefore the chromosome segregation throughout mitosis, independently of the cell origin or type. In order to determine whether this effect was significant after infection, we quantified the percentage of aberrant mitosis in non-infected and infected monolayers. We defined aberrant mitosis as those mitotic spindle problems in chromosome segregation, mislocated centrioles or an increased number of centrosomes. The results showed that only the bovine cell line BCEC exhibited a significant increase in the number of aberrant mitoses after infection (Fig. 3C). Human endothelial cells and fibroblasts displayed an increased percentage of aberrant mitosis, while epithelial cell mitosis was not affected by infection (Fig. 3C).

**Fig. 4:**
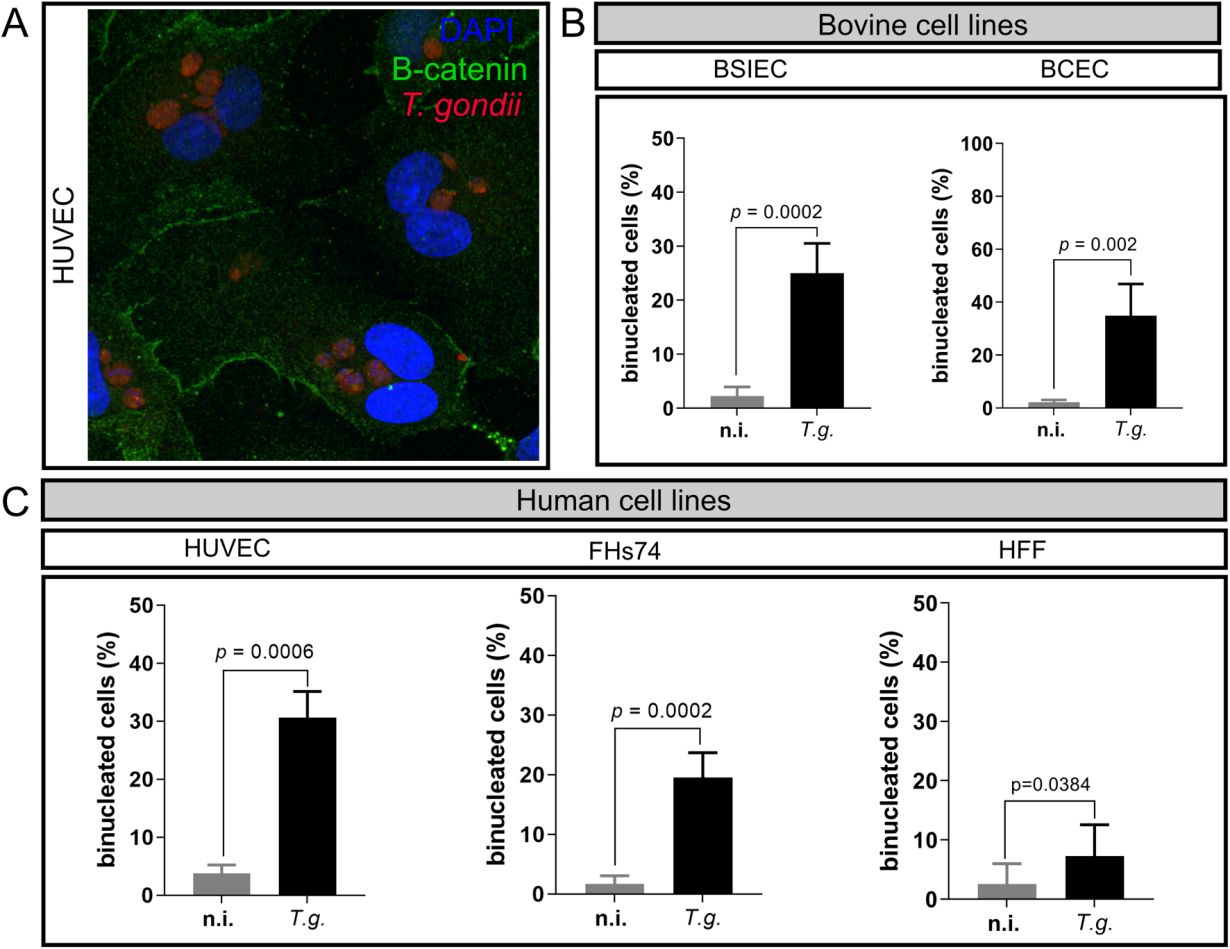
*Toxoplasma gondii* induces cytokinesis failure in human and bovine host cells. HUVEC, HFF, FHs74, BSIEC and BCEC were infected with *T. gondii* tachyzoites, and fixed 24 h p.i. with PFA and stained against DAPI (nuclear marker, blue), β-catenin (membrane marker, green) and *T. gondii* (red). As in the scheme, binucleated cells were those cells with more than one nucleus per cell (**A**). The total number of binucleated cells was normalized to the total number of cells counted and presented as a percentage in the graphs. The results showed that all tested cell lines increased the percentage of binucleated cells after infection with *T. gondii* (**B**, **C**). Graph bars represent the median ± SD of three biological replicates

Finally, we wanted to know whether the cytokinesis failure observed after *T. gondii* infection was cell-type or cell-origin-specific. Cytokinesis failure means a failure in cytosol division at the end of mitosis; therefore, we quantified the total number of cells displaying more than one nucleus per cell (binucleated cells; Fig. 4). Human and bovine cells were fixed 24 h p.i. and stained against *T. gondii* and DAPI, and the total number of binucleated cells was counted (Fig. 4). The results showed that all cells tested had increased percentages of binucleated cells, independently of the origin or cell type (Fig. 4). We consistently observed parasite-driven cytokinesis inhibition resulting in 18–34% of the host cells with more than two nuclei in all cell types (Fig. 4). Taken together, the current data suggest that *T. gondii*-induced chromosome segregation errors and cytokinesis failure are neither host species- nor host cell type-dependent. However, host cell cycle arrest was only observed in four of the five cell types tested in the current study, suggesting a parasite mechanism that might relate to the cell type.

## Conclusions

*Toxoplasma gondii* is an obligate intracellular parasite that is globally spread and causes severe health problems in humans and animals, such as abortion, severely affecting the progeny welfare [2]. Given that this parasite can infect almost any warm-blooded animal, the mechanism that *T. gondii* uses to develop inside the host cell is of interest to scientists worldwide. In the last year, increasing evidence has shown that *T. gondii* modulates the host cell cycle, but all studies involved only one or two types of cells, using different experimental approaches, MOIs or infection time. Also, some studies worked with immortalized or tumour cells, which are well known to lose the cell cycle checkpoint control that a primary cell has. Therefore, we wanted to determine whether *T. gondii* uses the control of the host cell cycle as a mechanism to ensure its intracellular development, independently of the species or cell type used as a host. In order to study the responses closest to a real infection scenario, we worked only with primary cells at a maximum of four passages after isolation. The results showed that the cell cycle of only one cell type (BCEC) was not influenced after infection. However, it is important to highlight that this cell line was isolated and monitored in our laboratory, with normal growth like the others, and normal intracellular development of *T. gondii* tachyzoites during infection as well. Therefore, the results might be analysed according to specific proteins that the parasite modulates in order to arrest the host cell cycle but that are not expressed in this cell type. Nevertheless, further experiments are needed to identify why *T. gondii* infection cannot control cell cycle progression in BCEC cells, but this is beyond the scope of the current work.

Regarding the mitosis and binucleated cell percentages, the results showed no differences between human and bovine cell lines. Therefore, we consider that both pathways controlled by *T. gondii* infection probably involved molecules or proteins that are not specific to a cell type or a determinate species. Similarly, it occurred with chromosome segregation errors, which were observed equally in all studied cell lines and species tested in the current study. Mitosis and cytokinesis need the cytoskeleton to function, specifically from the tubulin fibres which form the mitotic spindle or the midbody formation, respectively [22, 23]. Interestingly, *T. gondii* is well known to modulate the host cell tubulin cytoskeleton by relocating it around the parasitophorous vacuole (PV) [5]. After 1 h, infected cells showed a relocation of aster microtubules around the PV, and longer times of infection showed cells with multiple γ-tubulin foci suggesting critical microtubule dynamics in infected cells [5]. Therefore, our results can be explained by the control that *T. gondii* infection does on the host cell cytoskeleton and highlight that the failure cytokinesis process and the problems in the chromosome segregation are mechanisms that *T. gondii* uses in all infections, not depending on the host cell origin or cell type.

Our research indicates that *T. gondii* modulates host cell cycle progression, chromosome segregation and cytokinesis in primary cells independently of the host and cell type. Therefore, we suggest that these mechanisms represent a basal control of the parasite over the host cell. For future studies, it would be interesting to identify whether these *T. gondii-*induced phenotypes are all interconnected or if they represent a specific control that *T. gondii* separately exerts in pathways.

## Data Availability

The original contributions presented in the study are included in the article/Supplementary material.
